# Umbilical cord blood and cord tissue banking as somatic stem cell resources to support medical cell modalities

**DOI:** 10.1186/s41232-023-00311-4

**Published:** 2023-12-05

**Authors:** Tokiko Nagamura-Inoue, Fumitaka Nagamura

**Affiliations:** 1 Department of Cell Processing and Transfusion, Research Hospital, The Institute of Medical Science, The University of Tokyo, 4-6-1 Shirokanedai, Minato-ku, Tokyo, 108-8639 Japan; 2 IMSUT CORD, Research Hospital, The Institute of Medical Science, The University of Tokyo, Tokyo, Japan; 3 Division of Advanced Medicine Promotion, The Advanced Clinical Center, The Institute of Medical Science, The University of Tokyo, Tokyo, Japan

**Keywords:** Umbilical cord, Umbilical cord blood, Mesenchymal stromal cells, Extracellular vesicles, Regenerative medicine, Immunotherapy, Gene and cell therapy

## Abstract

Human umbilical cord blood (CB) and umbilical cord tissue (UC) are attractive sources of somatic stem cells for gene and cell therapies. CB and UC can be obtained noninvasively from donors. CB, a known source of hematopoietic stem cells for transplantation, has attracted attention as a new source of immune cells, including universal chimeric antigen receptor-T cell therapy (CAR-T) and, more recently, universal CAR-natural killer cells. UC-derived mesenchymal stromal cells (UC-MSCs) have a higher proliferation potency than those derived from adult tissues and can be used anon-HLA restrictively. UC-MSCs meet the MSC criteria outlined by the International Society of Gene and Cellular Therapy. UC-MSCs are negative for HLA-DR, CD80, and CD86 and have an immunosuppressive ability that mitigates the proliferation of activated lymphocytes through secreting indoleamine 2,3-dioxygenase 1 and prostaglandin E2, and the expression of PD-L2 and PD-L1. We established the off-the-shelf cord blood/cord bank IMSUT CORD to support novel cell therapy modalities, including the CB-derived immune cells, MSCs, MSCs-derived extracellular vesicles, biological carriers loaded with chemotherapy drugs, prodrug, oncolytic viruses, nanoparticles, human artificial chromosome, combinational products with a scaffold, bio3D printing, and so on.

## Background

Human umbilical cord blood (CB) has been a well-known source of hematopoietic stem cells (HSCs) for over 25 years. In the present day, CB transplantations are implemented more than 1200 per year in Japan (http://www.jdchct.or.jp/data/report/2022/). However, the number of CB transplantations (CBT) in European countries (EU) is decreasing, instead, the HLA-haploidentical allogeneic HSC transplantation has become popular in the EU rather than CBT. The reason why HLA-haploidentical relatives have become rapidly available sources of HSCs is that prevention methods for acute graft-versus-host disease (aGVHD), mainly post-transplant cyclophosphamide administration, resulting in the removal of alloreactive T cells in the patients [1]. CB is currently the optimal source for immunotherapy using activated T cells, regulatory T cells, and natural killer (NK) cells, with or without genetic modifications. Additionally, there has been an overwhelming interest in mesenchymal stromal cells (MSCs) for immunotherapy and regenerative medicine, although CBs are limited in volume to obtain an adequate number of MSCs. MSCs can be isolated from any tissue in the body, but currently, the major sources of MSCs include the bone marrow (BM), adipose tissue (AD), and umbilical cord (UC) [2]. Among these cell sources, human UC has been rapidly utilized as an abundant source of MSCs worldwide due to its ease of collection, noninvasive collection procedure, and categorization as biological waste at birth. Moreover, it is the youngest nonsenescent human tissue except for ES cells. This review focuses on CB- and UC-derived cells as a source of sustainable material for new modalities in gene and cell therapy.

## Characteristics and therapeutic potentials of CB and UC-MSCs

CB has been well-investigated and is known to include a relatively high potential of CD34-positive cells to be expanded, more naïve CD45RA-positive T cell ratios, and more potent suppressor function of regulatory T cells than adult peripheral blood [3, 4].

UC-MSCs meet the criteria of MSCs defined by The International Society for Gene and Cellular Therapy [5, 6]. First, they are plastically adherent when maintained in a standard culture medium supplemented with serum. Second, they are positive for CD105, CD73, and CD90 but negative for CD45, CD34, CD14 or CD11b, CD79α or CD19, and HLA-DR surface molecules. Third, MSCs cannot differentiate into adipocytes, chondrocytes, and osteoblasts in vitro.

Additionally, immunosuppressive abilities and tissue repair are the most important properties of MSCs for clinical use [7]. However, MSCs are activated to suppress the immune system only upon the inflammatory stimuli, including activated T cells, PHA-L, and IFN-γ. He et al. demonstrated that third-party UC-MSCs suppress the proliferation of CD4 and CD8-positive cells activated by allogeneic dendritic cells or inflammatory reagents [8]. Secreted factors such as indoleamine 2, 3-dioxygenase 1, and PGE2 are induced in UC-MSCs by the inflammatory environment and play a critical role in controlling excess immune system [9]. UC-MSCs constitutively express the PD-L2, while PD-L1 is induced in response to IFN-γ [10]. Furthermore, like BM- and AD-derived MSCs, MSCs are negative for HLA class II expression and the co-stimulatory surface antigens CD80 and CD86, which activate T cells [11]. UC-MSCs remain negative for HLA-DR even in the presence of IFN-γ, while HLA-DR on BM-MSCs can be induced upon IFN-γ stimuli [8]. As a result, these cells escape from activated T cells and are utilized in a non-HLA-restricted manner. Because of these anti-inflammatory properties, MSCs may be useful therapeutic candidates for the treatment of inflammatory disorders.

Another important characteristic of UC-MSCs is their ability to repair tissues. Kurtzberg et al. reported that autologous CB may be effective in mitigating the symptoms of cerebral palsy after birth. CD34-positive cells in the CB play a critical role in treating cerebral palsy, and clinical trials using autologous CB for hypoxic–ischemic encephalopathy (HIE) has been performed in Japan [12]. However, collecting adequate amounts of CB for therapeutic interventions is difficult. Recently, allogeneic UC-MSCs have become an attractive source to overcome the disadvantages of CB collection [13]. Causes of cerebral palsy include periventricular leukomalacia (PVL), periventricular hemorrhage, and HIE. However, a common mechanism of cerebral palsy is the early phase of inflammation caused by hypoxia, glucose depletion, and microglia dysfunction with reactive oxygen species, followed by neurogenic damage [13]. The excess inflammation and tissue damage in the pathological cascade are expected to be controlled by MSCs. We previously found that UC-MSCs migrate toward the injured site of the brain after tracking in the lungs, although MSCs do not engraft and disappear after 3 weeks of intravenous injection. UC-MSCs secrete neurotrophic factors such as brain-derived neurotrophic factor and hepatocyte growth factor, and attenuate mice brain injury [14, 15]. These characteristics of UC-MSCs described above are expected to contribute to the development of treatments in the fields of immunotherapy and regenerative medicine.

## Umbilical cord blood and cord bank

**Fig. 1:**
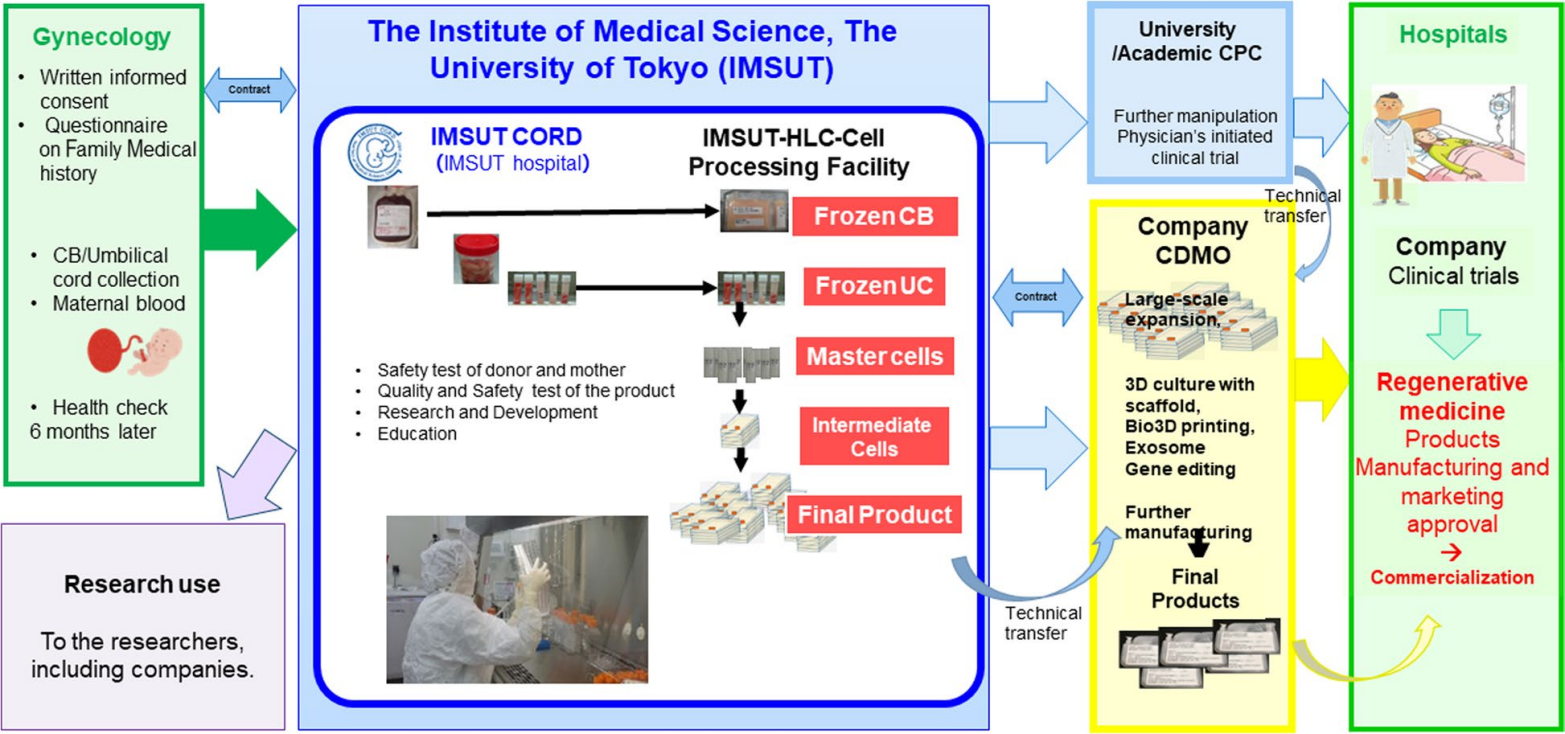
Overall flow of the off-the-shelf cord blood/cord bank, IMSUT CORD to support the clinical and research use

Establishing a stable supply system for CB- and UC-MSCs is critical for implementing regenerative and immunotherapies. For this purpose, The Institute of Medical Science, The University of Tokyo (IMSUT), has established the cord blood/cord bank IMSUT CORD as a new type of public biobank to supply “off-the-shelf” frozen CB, UC tissues, and UC-derived cells. Briefly, the IMSUT CORD collected both CB and UC data after obtaining informed consent from the guardian of the baby (Fig. 1). In addition to obtaining informed consent, questionnaires about the medical history, genetic history of the baby donor’s family, and history of the mother’s communicable disease risk behavior were collected. CB and UC were collected, and the mother’s blood was tested for infection. These documentation and tests in CB banks can also be referred to as UC banking, although additional infection-related tests for UC banking are strictly required. The collected CB and UC are transported from the hospitals to the CB/UC bank and the IMSUT CORD cryopreserves UC tissue [16] until obtaining confirmation that the baby exhibits healthy, normal development and the mother remains free from infection within at least 6 months after delivery.

The IMSUT CORD processes and freezes CB into frozen enriched cells, while freezes the UC tissue and cultures the frozen-thawed UC tissue into master cells, intermediate cell products, or final cell products, according to the physician’s or company’s requirements of cell modalities.

## Clinical trials using UC-MSCs for severe acute GVHD and others

Table 1 shows the reports of clinical trials for severe acute graft-versus-host disease (aGVHD) using UC-MSCs [10, 17]. Not like BM-MSCs, there are only two studies to treat severe aGVHD using UC-MSCs including the author’s one. Here, we introduce our physician-initiated clinical trial for severe aGVHD using allogeneic UC-MSCs (IMSUT-CORD) manufactured in serum-free medium and cryoprotectant from 2018 to 2020 in Japan [10]. In a phase I dose-escalation clinical trial, IMSUT-CORD was administered 1 × 10^6^ cells/kg or 2 × 10^6^ cells /kg intravenously twice per week over 2 cycles. Patients with no adverse effects, partial response (PR), or mixed response (MR) underwent up to two additional cycles. No severe adverse events were observed; at 16 weeks after the initial IMSUT-CORD infusion, one patient showed no response, while one showed MR, two showed PR, and three showed a complete response (CR). The overall response was 71.4% (90% CI 34.1–94.7%), while the overall survival was 85.7% (90% CI 62.2–100%). The continuous CR/PR rate was 100% for > 28 days after CR/PR, while the survival rate was 85.7% on day 100 (90% CI 62.2–100). The overall response rate (ORR) of 70% reported by Soder RP. et al. was similar that of ours. These high ORR and less toxicity show the necessity of conducting further clinical trials. Table 1 shows the reports of clinical trials using UC-MSCs. The overall response rate (ORR) of 70% reported by Soder RP., et al. was similar that of ours. These high ORR and less toxicity show the necessity of conducting further clinical trials.

**Table 1:** Clinical trials using allogeneic umbilical cord-derived mesenchymal stromal cells for acute GVHD

Authors	Disease	Phase	Patients number	Age (range) year	Cell number	Frequency, interval	Results	Adverse event
Soder RP., et al. [17] (2020)	Steroid-resistant aGVHD	1	10	35–73	2 × 10^6^ cells/kg, 10 × 10^6^ cells/kg	Day 0, day 7	ORR: 70% (4 CR, 3 PR). Day 100/180 survival: 90% /60%,	No AE
Nagamura-Inoue, T., et al. [10] (2022)	Steroid-resistant aGVHD	1	7	25–62	1 × 10^6^ells/kg, 2 × 10^6^/kg	Twice/week, for 2 weeks. 2 additional weeks for PR or MR patients with no SAE	ORR: 71.4% (3 CR, 2PR, 1 MR,1 NR), over 28 days continuous CR/PR: 100%	no SAE

All MSCs were injected intravenously

*Day 0* the first administration day

*AE* adverse event, *aGVHD* acute graft-versus-host disease, *CR* complete response, *MR* mixed response, *NR* no response, *ORR* overall response rate, *PR* partial response, *SAE* severe adverse event

Other implementations of clinical trials using IMSUT-CORD include phase I clinical trial for COVID-19-related acute respiratory distress syndrome (ARDS), phase I clinical trial for cerebral palsy, and phase II clinical for post-hematopoietic transplantation non-infectious pulmonary complication in Japan. While all three clinical trials were closed, the results have not been published yet.

In our COVID-19-related ARDS study, there are six publications in the world (Table 2) [18–23]. Among three phase II clinical trials for COVID-19-related ARDS, one showed an improvement in survival rate significantly [19], while two did not [18, 20].

**Table 2:** Clinical trials using allogeneic umbilical cord-derived mesenchymal stromal cells for COVID-19-related ARDS and cerebral palsy

Authors		Disease	Phase		Patients number		Age (range) year		Cell number	Frequency, interval	Results	Adverse event
COVID-19-related ARDS												
Monsel A., et al. [18] (2022)		Moderate to severe COVID-19-related ARDS	2b		MSC: 21, placebo: 24		64 (mean)		1 × 10^6^ cells/kg	Day 0, day 2 ± 1, day 4 ± 1	No efficacy of PaO2/FiO2-ratio and mortality	No SAE
Dilogo IH., et al. [19] (2021)		Critically ill COVID-19-related ARDS	2		MSC: 20, placebo: 20		< 40:4, 40–60: 8, > 60: 8		1 × 10^6^ cells/kg	One dose	Survival rate in UC-MSCs group was 2.5 times higher (*P* = .047), that in UC-MSCs group with comorbidities, 4.5 times higher. The length of stay in the intensive care unit and ventilator usage were not statistically significant	No AE
Gorman EA., et al. [20] (2023)		Moderate to severe COVID-19-related ARDS	2		MSC: 30, placebo: 29		58.4 (mean)		4 × 10^8^ cells	One dose	Improvement of pulmonary organ dysfunction: no difference	Safe and well tolerated
Lanzoni G., et al. [21] (2021)		Mild to severe COVID-19-related ARDS	1/2		MSC: 12, placebo: 12		58.6 (mean)		100 ± 20 × 10^6^ cells	Day 0, day3	Survival rate at day 28: MSC 91, placebo 42%, *P* = .015), SAE-free survival (*P* = .008), time to recovery (*P* = .03)	No SAE, no difference in AEs
Farkhad NF., et al. [22] (2022)		Mild to moderate COVID-19-related ARDS	1		MSC:10, control: 10		62.0 (mean)		1 × 10^6^ cells/kg	Day 0, day 2, day 4	Improvement of patients’ clinical and paraclinical parameters (leukocytosis, lymphopenia, thrombocytopenia, liver enzyme abnormalities)	no SAE
Shaz BH., et al. [23] (2023)		COVID-19-related ARDS	1		10		39–79		1 × 10^6^ cells/kg, maximum dose 10^8^ cells	Once a day for 3 days	Survival on day 28, 7 (70%)	No SAE
Neonatal encephalopathy												
Cotten CM., et al. [24] (2023)	Moderate to severe hypoxic ischemic encephalopathy, treated with hypothermia		1, pilot	6		36–41 weeks		1 or 2 doses of 2 × 10^6^ cells/kg/dose		First dose: during hypothermia, second dose: 2 months later	All babies survived, with average to low-average developmental assessment standard scores between 12 and 17 postnatal months	Well tolerated, low titer anti-HLA antibodies by 1 year of age: 5/6

All MSCs were injected intravenously

*Day 0* the first administration day

*AE* adverse event, *ARDS* acute respiratory distress syndrome, *COVID-19* coronavirus infectious disease emerged in 2019

In clinical trials for cerebral palsy, there is only one publication of phase I, a pilot study, for moderate to severe hypoxic ischemic encephalopathy (HIE) [24]. Six neonates with moderate (4) or severe (2) HIE were enrolled and received one dose of UC-MSCs during HIE and 2 received a 2nd dose 2 months later. All babies survived with average to low-average developmental assessment standards scores for ages between 12 and 17 postnatal months. No severe adverse events were reported in all papers, although 5/6 babies developed low titer anti-HLA antibodies.

As for post-hematopoietic transplantation non-infectious pulmonary complication, no report was found.

## CB and UC for exploring new cell modalities

### CB and UC-MSCs

Although the use of CB for hematopoietic stem cell transplantation has decreased recently in the world [1], new technologies have promoted the use of expanded CD34 + cells for HSCT [25], regulatory T cells to induce tolerance in HSCT [26, 27], universal chimeric antigen receptor-T cell therapy (CAR-T) [28], and universal CAR-NK cells [29–32] for hematological malignancies.

In addition to the clinical trials using UC-MSCs introduced in Tables 1 and 2, there are accumulating early-phase clinical trials using UC-MSCs [17, 20, 23, 33–35] for engraftment facilitation in HSCT for aplastic anemia [36], neurogenic injuries, diabetes mellitus (DM), heart and angioplasty, liver damage including liver cirrhosis, inflammatory bowel diseases such as Crohn’s disease and ulcerative colitis, prevention of acute rejection in renal transplantation, and collagen diseases [33]. The number of relevant clinical trials on the NIH clinical trial website (https://00m.in/HHVJP) has increased up to 95 trials, including complete, recruiting, and not-yet-recruiting status, as of the end of May 2023.

### UC-MSCs-derived extracellular vesicles (EVs)

EVs derived from MSCs might be expected to have effects comparable to those of their parental cells. When parental cells exert therapeutic potency, their EVs may carry the key functional molecules by priming [37, 38]. EV therapy is an emerging type of next-generation cell therapy, but properly testing the safety and efficacy of EVs is challenging. Recently, Rohde et al. proposed clinical testing for the manufacture and characterization of EVs derived from UC-MSCs [39]. The number of preclinical proof-of-concept reports using EVs derived from UC-MSCs is increasing. Chu M. et al. reported the result of a phase 1 clinical trial of nebulization of UC-MSCs derived exosome for patients with COVID-19 pneumonia [40]. The dose of exosome was the same amount of MSCs proportional to the patient’s body weight (1 × 10^6^ cells/kg), and the concentration of exosomes for nebulization ranged from 7.66e + 0.8 to 7.00e + 0.7 particles/ml based on NanoSight. Promoted the absorption of pulmonary lesions and reduced the duration of hospitalization for mild cases of COVID-19 pneumonia were observed with no adverse events.

Other clinical trials using exosomes from UC-MSCs listed on Clinicaltrials.gov are summarized in Table 3. Target diseases are various, such as COVID-19-related diseases, multiple organ dysfunction syndrome after surgical repair of acute type A aortic dissection liver cirrhosis, and retinitis. Out of six clinical trials, one is in a phase II/III study, and another one is in a phase II study. The exosome therapies may be developed rapidly in the near future.

**Table 3:** Clinical trials of exosome listed on ClinicalTrials.gov

Number	Principal Investigator	Sponsor	Disease	Phase	Patient number	Route of administration	Dose	Frequency, interval
NCT05808400	Jihui Du	Huazhong University of Science and Technology, China	chronic cough after COVID-19	1	Exosome: 40, Control: 40	iv	1 × 10^9^ particles/ml. × 5 ml	5 days, twice daily
NCT04356300	Liang-Wan Chen	Fujian Medical University, China	Multiple organ dysfunction syndrome after surgical repair of acute type A aortic dissection	N/A	60	iv	150 mg	Once a day for 14 days
NCT05871463	Behzad Hatami	Research Institute for Gastroenterology and Liver Diseases, Iran	Decompensated liver cirrhosis	2	15	iv	Final dose of 40 mg	3 weeks
NCT05413148	Kuddusi Erkılıç	TC Erciyes University, Turkey	Retinitis	2/3	135: MSC vs. exosome vs. placebo	Subtenon’s injection for single eye	Not described	One dose
NCT05787288	Xiaoying Huang	First Affiliated Hospital of Wenzhou Medical University, China	COVID-19 Pneumonia	early phase 1	240 (Nebulized extracellular vesicles (EV) vs. saline)	iv	1 × 10^9^ particles/ml × 5 ml	Twice a day for 5 days
NCT05387278	no information	Vitti Labs, LLC	Severe ARDS associated with COVID-19	1	20 (Exosome + MSC 10 vs. placebo 10)	iv	No information	No information

*COVID-19* coronavirus infectious disease emerged in 2019

### Other modalities

Although UC-MSCs differentiate into osteocytes less frequently than BM-MSCs, tissue engineering with scaffolds such as poly (D, L-lactide-co-glycolide) has been shown to facilitate UC-MSC osteogenesis in a mouse model [41].

Recently, Ikeguchi et al. reported the first clinical application of a Bio three-dimensional (3D) nerve conduit made from a spheroid of human fibroblasts [42] using a bio 3D printer to treat peripheral nerve injury [43], although not yet constructed with UC-MSCs.

UC-MSCs or UC-derived cells can be modified by gene transfer; however, this has not yet been achieved at the clinical level. Meshizuka et al. demonstrated that the CRISPR/Cas9- and AAV-mediated insertion of the beta2 microglobulin-HLA-G fusion gene protected UC-MSCs from allogeneic rejection in a GVHD setting in vitro [44].

MSCs migrate to the tumor microenvironment (TME) and promote tumor cell generation, mainly through the cross-talk of tumor parenchymal cells, tumor-associated fibroblasts, cytokines, and chemokines in the TME, secreting transforming growth factor-β and VEGF recruiting regulatory T cells. Owing to their accessibility, UC-MSCs may be modified and processed into effective biological carriers for loading with chemotherapy drugs, prodrugs, oncolytic viruses [45–47], nanoparticles, and human artificial chromosomes [48].

Many studies have been conducted to determine the efficacy of proof-of-concept in treating unmet diseases using CB and UC-MSCs, or their new modality cells.

## Conclusions

Both human umbilical CB and UC can serve as effective “off-the-shelf” sustainable sources for gene and cell therapies in immunotherapies and regenerative medicine.

## Data Availability

The review does not include patient data; however, data and materials related to banking are made available. Requests may be sent to crc-bank@ims.u-tokyo.ac.jp.

## Abbreviations

AD: Adipose tissue
aGVHD: Acute graft-versus-host disease
ARDS: Acute respiratory distress syndrome
BM: Bone marrow
CAR-T: Chimeric antigen receptor-T cell therapy
CB: Umbilical cord blood
CR: Complete response
DM: Diabetes mellitus
EVs: Extracellular vesicles
HIE: Hypoxic–ischemic encephalopathy
HSCs: Hematopoietic stem cells
HSCT: Hematopoietic stem cell transplantation
IMSUT CORD: The Institute of Medical Science cord blood/cord bank
MR: Mixed response
MSCs: Mesenchymal stromal cells
NK: Natural killer
PR: Partial response
TME: Tumor microenvironment UC, umbilical cord
UC: Umbilical cord tissue
UC-MSC: Umbilical cord-derived mesenchymal stromal cells
